# Pain and high-impact pain in community-dwelling older adults in Australia and relation to sociodemographic and health-related factors, including physical disability, psychological distress, and quality of life

**DOI:** 10.1186/s12916-026-04642-0

**Published:** 2026-01-22

**Authors:** Grace Joshy, Saman Khalatbari-Soltani, Kay Soga, Melonie Martin, Sinan Brown, Fiona M. Blyth, Emily Banks

**Affiliations:** 1 National Centre for Epidemiology and Population Health, College of Law, Governance, and Policy, The Australian National University, Australian Capital Territory, Canberra, Australia; 2 Sydney School of Public Health, Faculty of Medicine and Health, The University of Sydney, Sydney, NSW Australia

**Keywords:** Pain, High-impact pain, Physical functioning, Psychological distress, Quality of life, Self-rated health, Disability, Person-centred outcomes, Australia, Population-based

## Abstract

**Background:**

Chronic pain is common and debilitating and significantly impacts quality of life (QoL). However, large-scale population-based evidence on general bodily pain, pain sufficient to impact daily life (high-impact pain), and their relation to sociodemographic and health-related outcomes is limited.

**Methods:**

Self-administered questionnaire data from the 45 and Up Study (Wave-2, 2012–2015), an Australian population-based cohort study, were used to estimate the prevalence of general and high-impact pain. Modified Poisson regression estimated age- and sex-adjusted prevalence ratios (PRs) quantified their relation to sociodemographic, behavioural, and health characteristics, as well as physical functioning, psychological distress, QoL, and self-rated health.

**Results:**

Overall, the study included *n* = 142,313 participants. Among them, 31% reported moderate-to-severe bodily pain, and 13% reported high-impact pain. High-impact pain was more common among females (14.3% versus 12.0% in males; PR = 1.24 [1.21–1.28]), older adults (18.8%, PR = 1.73 [1.66–1.79] for age ≥ 80 years; 14.2%, PR = 1.29 [1.26–1.33] for 65–79 years; versus 11.2% for 45–64 years), those least physically active (versus most active), currently smoking (versus never-smoking), obese, or had chronic health conditions. The prevalence of high-impact pain was markedly higher among those with lower education levels, lower household income, physical disability, psychological distress, or low QoL. Similar patterns were observed for bodily pain, although associations were weaker. Consistently, people reporting greater high-impact pain and bodily pain were substantially more likely to experience severe physical functioning limitations, moderate-to-high psychological distress, and poor/fair self-rated health and QoL compared to people without such pain. For example, 47.2% of the 16,825 people with high-impact pain had severe physical limitations versus 4.0% of 30,748 people without impactful pain (PR = 10.35 [9.78–10.95]); among those with high-impact pain, 40.5%, 36.5%, and 26.7%, respectively, had moderate-to-high psychological distress (PR = 4.61 [4.43–4.80]), poor/fair self-rated health (PR = 8.64 [8.16–9.14]), and poor/fair QoL (PR = 8.73 [8.16–9.34]).

**Conclusions:**

Bodily pain sufficient to interfere with daily life affects around one-in-eight older community-dwelling participants. People of lower socioeconomic position and those with health problems, particularly physical disability, are more likely to experience high-impact pain. Among those experiencing high-impact pain, around half have severely reduced physical functioning or high psychological distress, and a quarter report poor/fair QoL.

**Supplementary Information:**

The online version contains supplementary material available at 10.1186/s12916-026-04642-0.

## Background

Chronic pain is common, affecting more than 30% of people worldwide [1, 2]; it is closely associated with reduced quality of life (QoL) and imposes a significant socioeconomic burden on individuals and society [1, 2]. Pain and related conditions, including low back pain, headaches, neck pain, and osteoarthritis, are leading causes of years lost to disability globally [3]. In 2018, 3.24 million Australians were estimated to be living with chronic pain, with the majority (56%) experiencing restrictions on the activities they are able to undertake in their daily lives [4].

The prevalence of pain varies across countries and communities, ranging from 10 to 50% [5]. Studies vary in terms of the type of pain investigated, assessment of severity and/or impact of pain, and the study population, with the majority being from the USA, UK, and Europe [5]. In general, pain is more common among females, middle-aged and older people, those with chronic disease, and those of low socioeconomic position, as indicated by lower levels of education, and smoking [5]. The prevalence of chronic pain is high in older age groups, driven by aging itself, disability, higher incidence of diseases, slower healing, and poorer recovery from acute injury [6]. Older adults are particularly vulnerable due to an increased risk for suffering from bothersome pain and reduced ability to cope or manage pain.

For people living with pain, the extent to which it interferes with daily life is of central importance. However, evidence on this is limited—especially in the general population. Most studies to date have focused on the nature of pain (e.g., chronic widespread pain [7]; transition from regional to widespread chronic pain [8, 9]), on single indicators of socioeconomic position [10, 11], demographic [12], or health-related factors [13, 14] as exposures, on specific population groups [15, 16], and many are limited by small sample sizes. Although pain has been shown to be associated with mental health conditions, disability, and QoL [17–19], evidence is particularly limited on pain that impacts daily life and how it relates to person-centred outcomes – outcomes that matter to individuals and are key determinants of the ability to live rich and meaningful lives [20]. Since debilitating pain has important social, economic, and health consequences, multidisciplinary approaches to pain management, enabling meaningful contributions from individuals to family, work, and social life, are a public health priority.

This study aimed to quantify the prevalence of bodily pain and pain sufficient to impact daily activities (high-impact pain) in a population-based cohort study of older adults, according to sociodemographic, behavioural, and health characteristics. A further aim was to examine the relationship of pain to adverse person-centred outcomes, including physical functioning limitations, psychological distress, self-rated health, and self-rated QoL.

## Methods

### Study participants

The Sax Institute’s 45 and Up Study is a cohort study of 267,357 men and women aged 45 years or over, randomly sampled from the general population of New South Wales (NSW), Australia, using the Services Australia Medicare enrolment database. People from regional and remote areas based on the Accessibility and Remoteness Index of Australia and those aged 80 years or over were oversampled. Individuals joined the study by completing postal questionnaires between 2005 and 2009 and consenting to long-term follow-up through repeated surveys and linkage of their data to other population health databases [21]. Details of the 45 and Up Study are described elsewhere [21].

The follow-up to the 45 and Up Study (Wave 2) was undertaken from 2012 to 2015, where eligible participants completed a questionnaire gathering data on health and lifestyle changes, as well as additional specific data on physical health and wellbeing. Participants who had requested not to be contacted further or were deceased (ascertained through linkages to death registries) were not included. A total of 142,548 participants completed the follow-up survey. Questionnaire data included comprehensive self-reported information including demographic factors, doctor-diagnosed health conditions, pain, functional capacity, mental health and self-rated health, QoL, height, weight, smoking, alcohol intake, and physical activity (Additional file 1: Tables S1–S2) [22, 23].

Following the exclusion of participants with study withdrawal requests, the original follow-up survey data from the Sax Institute consisted of 142,412 participants. We excluded participants with invalid baseline or follow-up questionnaire dates (*n* = 95, 0.07%) or baseline age under 45 (*n* = 4, 0.003%). The analysis dataset consisted of 142,313 individuals.

### Outcomes

Main outcomes were bodily pain and its impact on daily life, based on two questions: (1) “How much bodily pain have you had during the past 4 weeks?”, followed by response options of none, very mild, mild, moderate, severe, and very severe; (2) “During the past 4 weeks, how much did pain interfere with your normal work (including both work outside the home and housework)?”, followed by response options of not at all, a little bit, quite a bit, moderately, and extremely. In binary classification, participants were considered to have bodily pain if they answered ‘moderate’, ‘severe’ or ‘very severe’. Participants were classified as having high-impact pain (at least ‘mild’ pain and ‘moderate’ or ‘extreme’ pain interference), bothersome pain (‘moderate’ or ‘severe’ pain and ‘a little’ pain interference), low-impact pain (‘moderate’ or ‘severe’ pain without any pain interference (‘not at all’) or ‘mild’ pain with ‘a little’ pain interference) or no impact of pain (no pain or pain interference) (Additional File 1: Tables S1, S3). From this, we also created a binary classification for high-impact pain (yes/no).

Adverse person-centred outcomes included severe physical functioning limitations, moderate or high psychological distress, poor or fair self-rated health, and poor or fair QoL (Additional File 1: Table S2). Physical functioning limitations were assessed using the Medical Outcomes Study Physical Functioning (MOS-PF) score [24] eliciting self-reported data on limitations in the ability to perform moderate and vigorous physical activities and tasks such as lifting or carrying shopping; climbing stairs; walking; bending, kneeling, or stooping; and bathing or dressing. The MOS-PF is a valid and reliable measure of physical functioning [25], with a lower score indicating more severe functional limitation. Scores ranged from 0 to 100 and were categorised as severe (0 to < 60), moderate (60 to < 90), minor (90 to < 100), or no (100) limitations. Psychological distress was assessed using the Kessler-10 (K10), a validated measure of non-specific symptoms of psychological distress [26]. Respondents indicated the frequency of symptoms experienced in the past 4 weeks, from 1 ‘none of the time’ to 5 ‘all of the time’. Scores ranged from 10 (no distress) to 50 (severe distress) [27] and were categorised as low (≤ 15), moderate (16 to 21), or high (22 to 50) distress. Self-rated health and QoL were based on the question, “In general, how would you rate your overall health/quality of life?”, followed by response options of excellent, very good, good, fair, and poor.

### Sociodemographic, behavioural, and health-related factors

Sociodemographic characteristics included age, sex, education (no school certificate, certificate/diploma/trade, university degree), annual household income (< 20 K, 20–40 K, 40–70 K, ≥ 70 K), private health insurance, region of residence, and country of birth (Australian-born, not Australian-born) (Additional File 1: Table S2). Age at follow-up survey was categorised as 45–64 years; 65–79 years; and ≥ 80 years. The region of residence (derived from the address) was categorised as major city, inner regional, outer regional, and remote/very remote.

Health and behavioural characteristics included body mass index (BMI (kg/m^2^), 15 to < 18.5, 18.5 to < 25, 25 to < 30, and 30–50), physical activity (tertiles of physical activity sessions per week weighted for intensity), smoking status (never/past/current smoker), and number of alcoholic drinks per week (0, 1–14, ≥ 15 drinks per week), and chronic health conditions (based on responses to the question “has a doctor ever told you that you have…”), followed by a tick list of conditions (Additional File 1: Table S2); we considered cardiovascular disease (high blood pressure, stroke or blood clot), cancer, diabetes, Parkinson’s disease, asthma, osteoarthritis, depression, and anxiety. Individual chronic conditions (yes/no), as well as the number of chronic conditions with a known relationship to pain (cancer, diabetes, osteoarthritis, anxiety, and depression) grouped as none, one, or two or more, were considered [28]. Sensitivity analysis considered the number of chronic conditions, grouping three conditions with no specific relationship to pain (cardiovascular disease (CVD), Parkinson’s disease, and asthma), as well as grouping all eight conditions irrespective of the relation with pain. Regular medications were based on responses to the question, “Have you taken any medications for most of the last 4 weeks?”, followed by a tick list of medications, which included ‘paracetamol without codeine’, ‘paracetamol with codeine’, and ‘aspirin for other reasons’. Indicators of physical and mental health included physical functioning limitations, psychological distress, self-rated health, and self-rated QoL. Lower back pain (yes/no) was assessed using responses to “In the past 4 weeks, have you had pain in your lower back?”.

### Statistical methods

We excluded participants with missing data on bodily pain (*n* = 3730; 2.6%) and high-impact pain (*n* = 3711; 2.6%) from the corresponding analyses. After logical imputation and backfilling for K10 and MOS-PF scores, we excluded those with missing data on each adverse person-centred outcome from the corresponding analyses.

Descriptive statistics summarised levels of bodily pain and high-impact pain in the study population, overall and according to sociodemographic and health-related characteristics. We quantified variations in levels of pain across these characteristics; modified Poisson regression models estimated prevalence ratios (PRs) and 95% confidence intervals (CIs) adjusted for age and sex (where applicable). Further statistical adjustments were not done as the objective was to compare prevalences rather than establish causality.

To quantify variation in adverse person-centred outcomes according to the level of pain experienced by the individual, adjusted PRs for severe physical functioning limitations, high psychological distress, poor/fair QoL, and poor/fair self-rated health were estimated by levels of bodily pain (no pain, very mild or mild pain, and moderate, severe or very severe pain) and impact of pain (no impact, low-impact, bothersome, high-impact).

## Results

The study sample included 142,313 participants; 54% of participants were aged > 65 years, 55% were women, and 78% were Australian-born (Table 1).

**Table 1:** Characteristics of the study population

	***N***	**%**
**Total participants**	**142,313**	**100%**
Age group (years)		
45–64	66,580	47%
65–79	59,236	42%
≥ 80	16,497	12%
Male	63,816	45%
University degree	40,590	29%
Annual household income $70,000 or more	41,720	29%
Private health insurance (hospital/DVA)	100,827	71%
Residing in major city	67,562	47%
Australian-born	110,846	78%
Body mass index, kg/m^2^		
Underweight (15 to < 18.5)	1599	1%
Obese (30 to 50)	31,084	22%
Highest physical activity tertile	37,988	27%
Current smoker	85,211	60%
≥ 15 alcoholic drinks per week	18,898	13%
Self-reported chronic condition		
Cardiovascular disease	31,852	22%
Cancer	22,264	16%
Diabetes	14,203	10%
Parkinson’s disease	1076	1%
Asthma	17,231	12%
Osteoarthritis	26,779	19%
Depression	21,123	15%
Anxiety	15,380	11%
None of the above	54,557	38%
Medications		
Paracetamol without codeine	37,445	26%
Paracetamol with codeine	9032	6%
Aspirin for non-cardiac reasons	6287	4%
None of the above	96,463	68%
Severe physical functioning limitations (MOS-PF score < 60)	19,503	14%
Moderate/high psychological distress (K10 score 16–50)	26,803	19%
Poor/fair self-rated health	17,874	13%
Poor/fair quality of life	11,807	8%
Received cancer treatment in last month	4518	3%

**Fig. 1:**
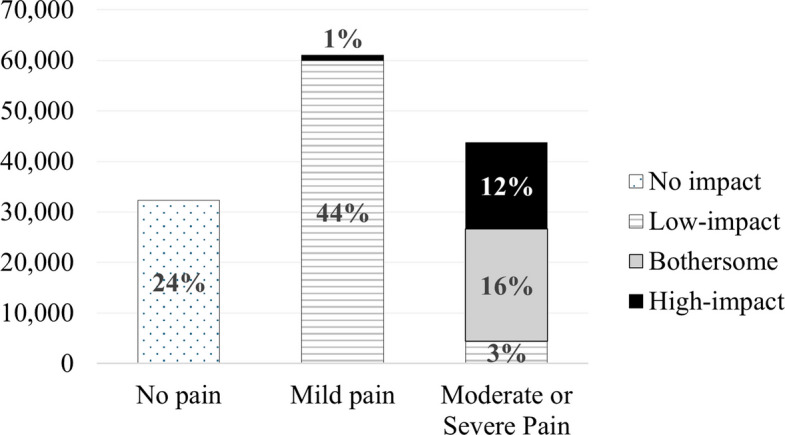
The distribution of pain and its impact in the study population

Overall, nearly a quarter of the study population (24%) reported no pain, 45% reported mild pain, and nearly one-third (31%) reported moderate/severe bodily pain (Fig. 1). The impact of pain on day-to-day activities was low for 47%, bothersome for 16%, and high for 13% of participants. While there is no additional information on the location of pain, at least 50% of those reporting any bodily pain or its impact also reported lower back pain, while 14% of those not reporting bodily pain in general reported lower back pain (Additional File 1: Fig. S1).

### Variations in pain and its impact according to sociodemographic and health-related factors

**Fig. 2:**
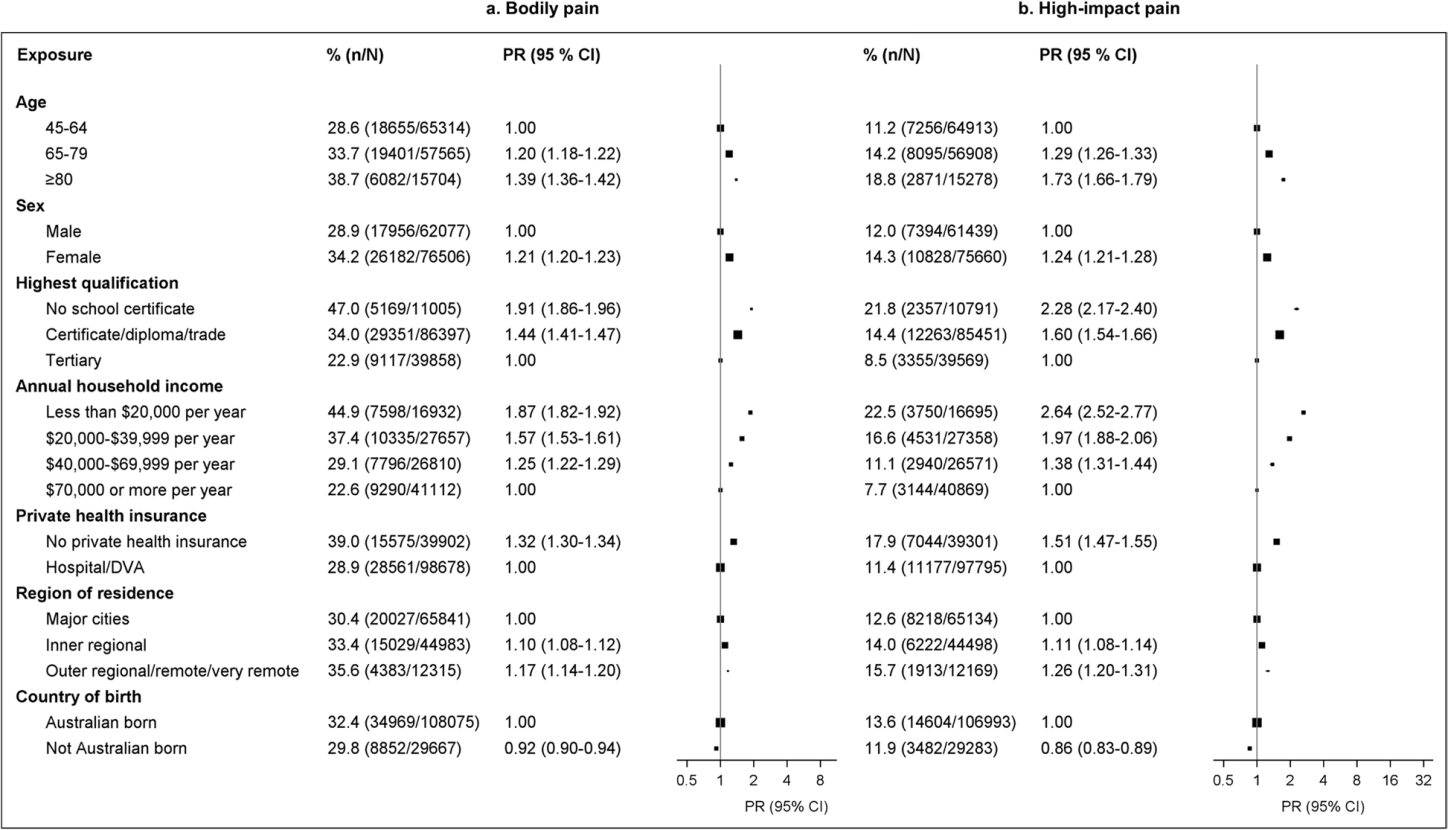
Prevalence of bodily pain and high-impact pain by sociodemographic characteristics. **a** Bodily pain. **b** High-impact pain. A total of 138,583 participants with valid data on bodily pain and 137,099 participants with valid data on the level of impact of pain contributed to the corresponding analyses. Models are adjusted for age (continuous variable) and sex, where applicable. Missing data in educational qualification, region of residence, and country of birth (0.95%, 11%, and 0.61%, respectively) were included as separate categories in the corresponding models; there were no missing data on age or sex. CI: confidence interval; PR: prevalence ratio adjusted for age and sex (where applicable)

**Fig. 3:**
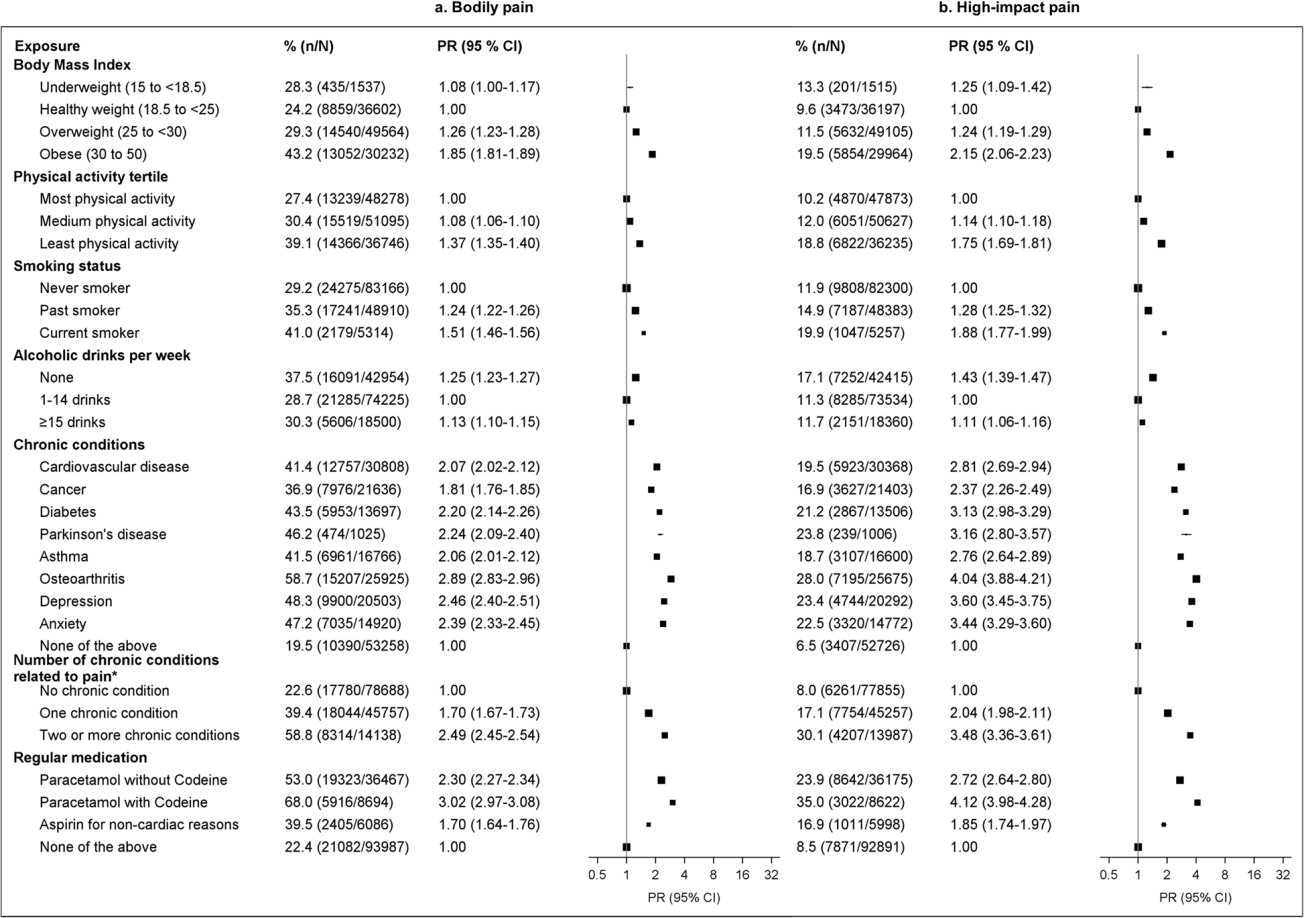
Prevalence of bodily pain and high-impact pain by health and behavioural factors. **a** Bodily pain. **b** High-impact pain. ^*^Chronic conditions with a known relationship to pain—cancer, diabetes, osteoarthritis, anxiety, and depression. Models are adjusted for age (continuous variable) and sex. Missing data in risk factors were included as separate categories in the corresponding models. The proportion of missing data in risk factors was less than 2%, except for BMI (14.9%); there were no missing data in chronic diseases and regular medications, as that information was collected using tick boxes. CI: confidence interval; PR: prevalence ratio adjusted for age and sex

High-impact pain, and to a lesser extent bodily pain, was more common among older adults, women, those with low education, low household income, no private health insurance, and those living in outer regional/remote/very remote areas (Fig. 2; Additional File 1: Fig. S2; Additional File 1: Table S4), compared to other cohort members. Prevalence of high-impact pain increased steadily with increasing BMI and was higher among the least physically active (versus most active), and those currently smoking (versus never smoking) (Fig. 3). High-impact pain was more common in those reporting alcohol consumption of ≥ 15 drinks per week (17.1%) and was slightly elevated in those reporting no alcohol consumption (11.7%) versus moderate consumption (11.3%). Those diagnosed with any of the eight chronic conditions considered were around three to four times more likely to report high-impact pain, with prevalence ranging from 16 to 28% for specific conditions versus 6.5% for those with none of those conditions (Fig. 2; Additional File 1: Table S5). Considering various combinations of chronic conditions, high-impact pain varied by specific conditions individuals were diagnosed with but was much more common among those with multimorbidity (Additional File 1: Table S6). Prevalence of bodily pain and high-impact pain increased with the number of chronic conditions (Fig. 3; Additional File 1: Table S7); among people with two or more conditions with a known relationship to pain (osteoarthritis, diabetes, anxiety, depression, cancer), 58.8% reported bodily pain and 30.1% reported high-impact pain. Reported use of paracetamol with and without codeine was associated with a higher prevalence of pain outcomes. Similar patterns across population groups were observed for both high-impact pain and bodily pain, although stronger socioeconomic gradients, as well as stronger associations with adverse physical and mental health outcomes were observed for high-impact pain.

**Fig. 4:**
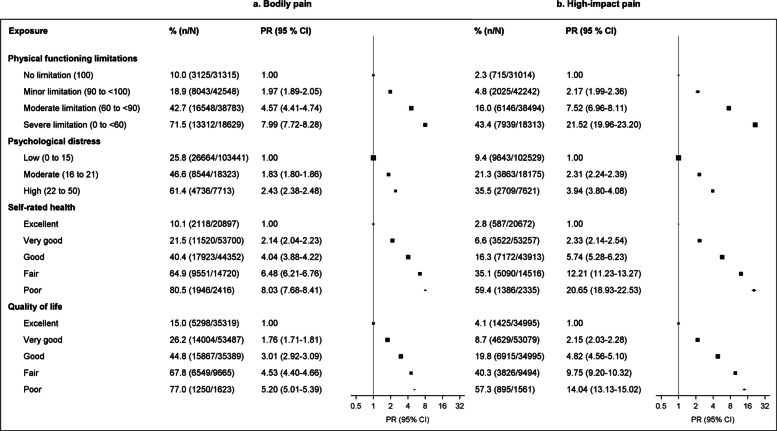
Prevalence of bodily pain and high-impact pain among participants by levels of physical functioning, psychological distress, self-rated health, and quality of life. **a** Bodily pain. **b** High-impact pain. Models are adjusted for age (continuous variable) and sex. Missing data in risk factors (1–7%) were included as separate categories in the corresponding models. CI: confidence interval; PR: prevalence ratio adjusted for age and sex

Compared to people with no physical functioning limitations, the prevalence of high-impact pain was two-fold in those with minor physical functioning limitations (4.8% versus 2.3%, PR = 2.17 [1.99–2.36]) and 21-fold in those with severe limitations (43.4%, PR = 21.52 [19.96–23.20]) (Fig. 4). Similarly, the prevalence of high-impact pain was 20-fold in those with poor versus excellent self-rated health (59.4% versus 2.8%, PR = 20.65 [18.93–22.53]), 14-fold in those with poor versus excellent QoL (57.3% versus 4.1%, PR = 14.04 [13.13–15.02]), and nearly four-fold in those with high versus low psychological distress (35.5% versus 9.4%, PR = 3.94 [3.80–4.08]). Bodily pain was much more common than high-impact pain in all these groups, with similar patterns but attenuated PRs observed (Fig. 4).

### Prevalence of adverse person-centred outcomes according to levels of bodily pain and impact of pain

**Fig. 5:**
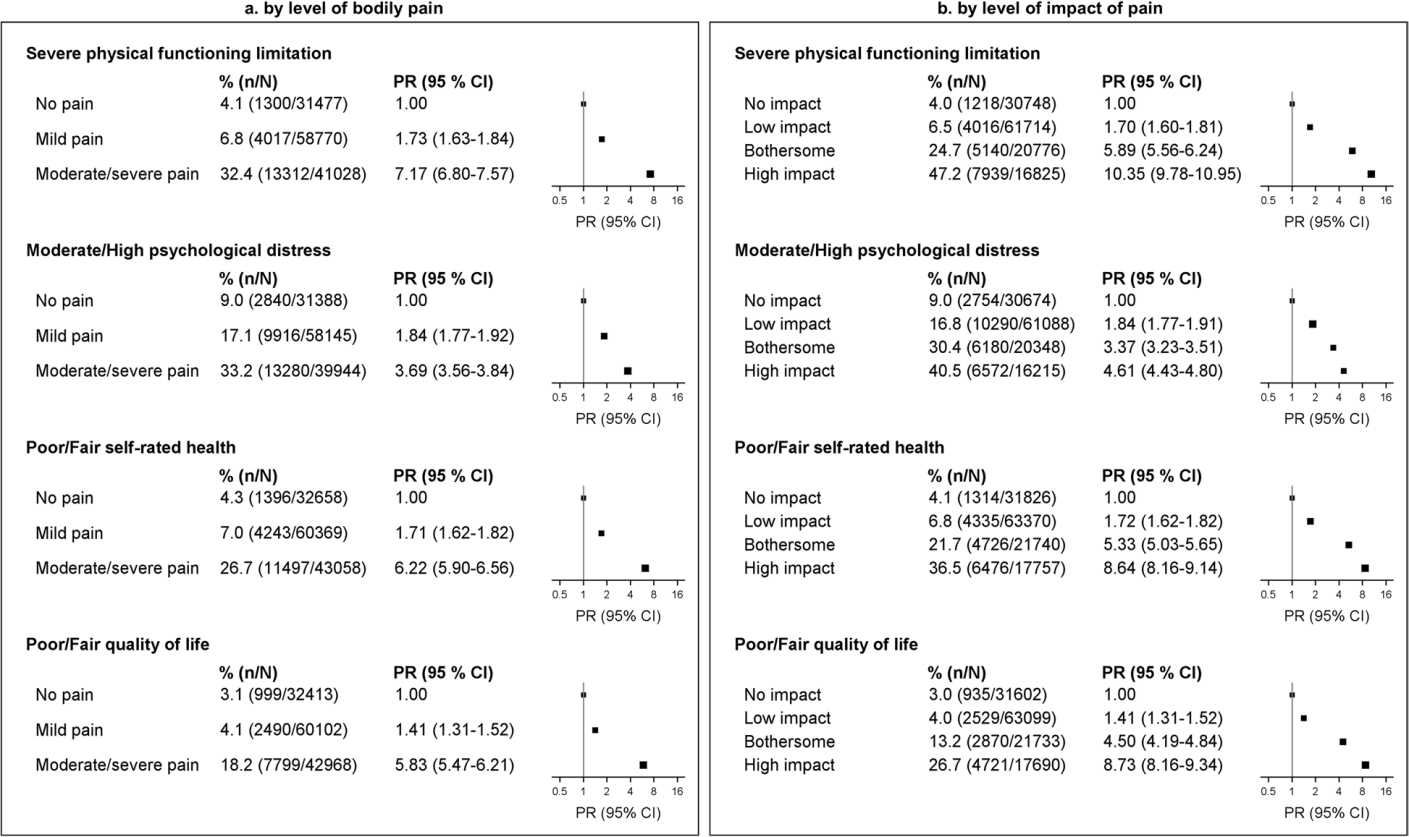
Prevalence of severe physical functioning limitation, moderate/high psychological distress, poor/fair self-rated health, and poor/fair QoL according to levels of bodily pain and impact of pain. **a** By level of bodily pain. **b** By level of impact of pain. Models are adjusted for age (continuous variable) and sex. CI: confidence interval; PR: prevalence ratio adjusted for age and sex. Of the 138,583 participants with valid data on bodily pain, those with missing data on each additional outcome variable were excluded from the corresponding analyses: 7308 for physical functioning limitations, 9106 for psychological distress, 2498 for self-rated health, and 3100 for quality of life. Of the 137,099 participants with valid data on high-impact pain, those with missing data on each additional outcome variable were excluded from the corresponding analyses: 7036 for physical functioning limitations, 8774 for psychological distress, 2406 for self-rated health, and 2975 for quality of life

Levels of adverse person-centred outcomes—severe physical functioning limitations, moderate/high psychological distress, and poor/fair self-rated health and QoL— increased with increasing levels of pain and its impact (Fig. 5). For instance, 47.2% of people with high-impact pain had severe physical limitations, compared to 4.0% of those reporting no impact of pain on their day-to-day activities (PR = 10.35 [9.78–10.95]); 40.5%, 26.7%, and 36.5%, respectively, had moderate/high psychological distress (PR = 4.61 [4.43–4.80]), poor/fair QoL (PR = 8.73 [8.16–9.34]), and poor/fair self-rated health (PR = 8.64 [8.16–9.14]). Prevalence of adverse outcomes was four-to-seven-fold in those with moderate/severe bodily pain compared to those reporting no bodily pain.

## Discussion

This large population-based study of over 140,000 middle-aged and older Australians found that pain is common, with around one-third reporting moderate/severe bodily pain and around one-in-eight reporting high-impact pain. Older adults, women, those with lower education and income, and those living in regional or remote areas are more likely than others to experience pain, reflecting a strong socioeconomic gradient. People born outside of Australia reported lower levels of pain than Australian-born participants. Pain was also more common among those with lower levels of physical activity, current smoking, high alcohol consumption or abstinence, chronic conditions, higher BMI, and use of over-the-counter medications for pain relief.

Our findings suggest a close, complex, and potentially bi-directional relationship between pain and adverse person-centred outcomes. Prevalence of bodily pain and high-impact pain increased substantially with increasing levels of adverse person-centred outcomes. At the same time, levels of adverse person-centred outcomes increased markedly with increasing levels of bodily pain and impact of pain. Among those with the most adverse person-centred outcomes (severe physical functioning limitations, high psychological distress, poor self-rated health, and poor QoL), at least 61–81% reported bodily pain and 36–59% reported high-impact pain; PRs were 2-to-22-fold compared with participants not reporting those issues. Among those with the highest levels of pain (moderate/severe bodily pain or high impact of pain), around 27–47% reported severe physical functioning limitations, moderate/high psychological distress, or poor/fair self-rated health, and 18–27% reported poor/fair QoL; PRs were 4-to-10-fold compared with participants not experiencing pain or impactful pain.

### Strengths and limitations

To our knowledge, this is the first Australian and largest study to quantify comprehensively the prevalence of bodily pain and the impact of pain, and to investigate high-impact pain in relation to sociodemographic, behavioural, and health-related factors. This study also adds to the evidence base on the relation of adverse person-centred health outcomes to pain and the impact of pain. The use of gradients on the impact of pain enabled a deeper understanding of the relationship between high-impact pain and person-centred outcomes, offering evidence to inform the development of multidisciplinary prevention and treatment strategies. In particular, this study highlights population groups with a higher prevalence of pain, including pain that affects their day-to-day functioning, as well as the broader problems experienced by those in pain.

The cross-sectional design of this study means caution should be applied to any causal interpretations. Temporal information on pain in relation to chronic conditions or person-centred outcomes was not available for this study. Our study population was randomly sampled from a whole-of-population database, representing about 10% of the entire NSW population in the target age group. The response rate was approximately 18%, consistent with similar cohort studies. Participants tend to be healthier than the general population in cohort studies, and attrition usually increases with every wave of follow-up. In general, nonparticipation and loss to follow-up tend to be more pronounced among the less advantaged and less healthy, leading to cohort studies often being an increasingly healthy, wealthy subpopulation [29]. Even when characteristics of follow-up samples are different to the initial sample, estimates on restricted samples can remain internally valid [30]. Hence, while the absolute estimates of pain prevalence may not be directly representative, the prevalence ratios (PRs), which are based on internal comparisons, are likely to be more generalisable [31]. We relied on survey data; most sociodemographic, health-related behaviours, and health-related determinants of pain and high-impact pain were self-reported. Of note, only data on country of birth were available (as a proxy for ethnicity), which warrants further studies on detailed ethnicity variables. Pain is based on self-report; linked clinical or qualitative interview data were not available to further understand issues such as how participants’ pain was addressed and whether they were currently able to access help from primary care providers.

### Findings in the context of the literature to date and possible mechanisms and implications

To our knowledge, there are no previous studies that have holistically evaluated a range of sociodemographic, behavioural, and health-related factors in relation to pain and high-impact pain in Australia. Most available evidence, in Australia and globally, focuses on chronic pain and does not consider pain alongside other person-centred outcomes—such as physical disability, psychological distress, and QoL. The experience of pain is dynamic, and measures used to assess pain vary [32], limiting comparability across studies; most measures consider duration, severity, and/or impact. The revised version of the graded pain scale [33] allows differentiation of mild, bothersome, and high-impact chronic pain based on the number of pain days in the past three months and activity limitation days due to pain. Measurement of high-impact pain in large cohort studies is an active area of research [34]. Although the graded pain scale was not available for this study, we were able to identify high-impact pain as recommended [32, 33], using activity limitations. Regardless of differences in study design, pain assessment method, and sample population, the associations we report are broadly consistent with prior studies, indicating that little has changed or improved over time. This highlights a need for strengthened policy attention and adequate resourcing to address modifiable risk factors for pain and a need to implement evidence-based, multidimensional prevention, and treatment strategies, with our larger, more comprehensive study providing greater certainty to support such actions.

Prevalence of pain and high-impact pain increased with increasing age, which agrees with previous findings that mostly report an increase in chronic pain prevalence at late middle age and then either a plateauing of prevalence estimates or continually increasing prevalence [9, 35]. Of note, some studies report no associations between increasing age and pain [17, 36–39] and some report decreasing prevalence among older adults relative to younger adults [15, 40, 41]. These inconsistencies suggest that differences in population characteristics (e.g., sociodemographic factors, coping strategies, and resilience) and methods used to measure pain may influence these associations.

Considering sociodemographic indicators of pain, our results are in agreement with previous literature, including a higher prevalence of pain among women [7–9, 17, 35], those with low educational level and income level [10, 38, 40–47], and those living in regional/rural areas [5, 37, 44]. We found differences in pain and high-impact pain by country of birth (lower prevalence among non-Australian-born compared to Australian-born); previous literature, mainly conducted in the USA, also reports differences in pain outcomes by ethnicity, finding mostly higher prevalence of pain among Native American and multiracial adults and lower prevalence among Asian Americans [48].

Prevalence of both pain and high-impact pain were significantly higher among those with higher BMI, lower levels of physical activity, past and current smokers, and those with no or high levels of alcohol consumption. These health-related and behavioural characteristics, including high BMI [35], low levels of physical activity [49, 50], and smoking [35, 51, 52], have been found to be associated with pain in previous studies. Existing evidence indicates that abstinence from alcohol is a risk factor, and moderate alcohol consumption is an apparent protective factor for pain [35]. Our findings, of higher proportions of participants with high alcohol consumption or abstinence reporting pain, might be due to the sick quitter effect, whereby former drinkers with underlying health conditions are included in the non-drinking group [53]. Additionally, given the cross-sectional nature of this study, these results should be interpreted with caution, and longitudinal studies are needed to establish the direction of these complex associations.

Similar to our findings, previous studies report a higher prevalence of pain among those with comorbid conditions, many of which are known to cause pain, including cancer, gastrointestinal, psychiatric/mood conditions [15, 28] Parkinson’s disease [54], cardiovascular diseases [55–58], and musculoskeletal conditions [15, 41, 45, 59, 60]. In addition, existing evidence indicates that multimorbidity is associated with pain [15, 28, 61]; although we looked at specific patterns of co-occurrence and the number of chronic conditions, we could not examine multimorbidity in detail considering time since diagnosis or severity. For example, 16% of participants reported being ever diagnosed with cancer, but only 3% had received cancer treatment in the past month. Our finding of a higher prevalence of bodily pain and high-impact pain among those using paracetamol with and without codeine likely reflects the common use of analgesic medications available at the time of the survey in pain management.

This paper provides new insights into the relation of pain to physical disability, psychological distress, and QoL, potentially reflecting bidirectional relationships with adverse person-centred outcomes likely contributing to and being consequences of pain. Our findings are generally consistent with the limited integrated evidence from previous studies. For instance, significant associations have been found between anxiety, depression, or poor mental health and pain [40, 52, 62–65]. Poor vitality and physical function have been shown to be associated with pain progression [66]. Poor self-rated health [52, 60] and low QoL [18, 67] have been reported as risk factors for pain, while poor mental health has been shown to be a predictor [66]. Our study found markedly greater adverse person-centred health outcomes in participants reporting moderate/severe bodily pain and bothersome or high levels of impact of pain. While pain has been reported as a key factor limiting activity/functioning [68, 69], there is limited evidence on how adverse person-centred outcomes vary according to increasing levels of pain. For example, we could identify only one study (*n* = 17,543, Australian population) reporting marked gradients in psychological distress and self-rated health by levels of chronic pain and interference with daily activities [40]. No previous studies have reported on adverse person-centred outcomes according to the level of impact of pain.

The study was not designed to determine the causal mechanisms through which sociodemographic, health-related, or behavioural factors contribute to the development of pain or its progression. The factors considered in this study were found to be associated with both pain and high-impact pain, which the biopsychosocial model of pain supports [70]. These factors are interconnected and the fact that the strongest relationships observed in this study were between physical ill health/disability and pain suggests this as a potential underlying mechanism. For instance, demographic factors (e.g., age, sex, ethnicity, geographical context) and socioeconomic position (e.g., low education and income levels) are major factors that influence health, the pain experience, and pain-related outcomes. Further research with more detailed analyses considering temporality is needed to evaluate causal relationships.

## Conclusions

Bodily pain that has a high impact on an individual’s ability to carry on with daily life affects around one in eight older community-dwelling participants. People of lower socioeconomic position and those with health problems, particularly physical disability, are more likely than others to experience high-impact pain. Among those experiencing high-impact pain, around half have severely reduced physical functioning and high psychological distress, and a quarter report poor/fair QoL. Findings inform prevention and management of pain and indicate that access to high-quality pain management would substantially improve outcomes.

## Supplementary Information

Additional file 1.

## Data Availability

This research was completed using data collected through the 45 and Up Study (www.saxinstitute.org.au). The study questionnaire is available at https:/www.saxinstitute.org.au/our-work/45-up-study/questionnaires. Data supporting the findings from this study are available from the Sax Institute. Restrictions apply to the availability of these data, which were used under license for the current study, and so are not publicly available. Data are available from the authors upon reasonable request and with permission of the Sax Institute (www.saxinstitute.org.au) and the NSW Department of Health.

## Abbreviations

BMI: Body mass index
CI: Confidence interval
CVD: Cardiovascular disease
K10: Kessler-10
MOS-PF: Medical Outcomes Study Physical Functioning
NSW: New South Wales
PR: Prevalence ratio
QoL: Quality of life

## References

[1] Cohen SP, Vase L, Hooten WM. Chronic pain: an update on burden, best practices, and new advances. Lancet. 2021;397:2082–97. 34062143 10.1016/S0140-6736(21)00393-7

[2] Mills SEE, Nicolson KP, Smith BH. Chronic pain: a review of its epidemiology and associated factors in population-based studies. Br J Anaesth. 2019;123:e273–83. 31079836 10.1016/j.bja.2019.03.023 PMC6676152

[3] Vos T, Lim SS, Abbafati C, Abbas KM, Abbasi M, Abbasifard M, et al. Global burden of 369 diseases and injuries in 204 countries and territories, 1990–2019: a systematic analysis for the Global Burden of Disease Study 2019. Lancet. 2020;396:1204–22. 33069326 10.1016/S0140-6736(20)30925-9 PMC7567026

[4] Deloitte Access Economics. The cost of pain in Australia. Canberra, Australia: Deloitte Australia; 2019. Available from: https://www2.deloitte.com/au/en/pages/economics/articles/cost-pain-australia.html.

[5] Zimmer Z, Fraser K, Grol-Prokopczyk H, Zajacova A. A global study of pain prevalence across 52 countries: examining the role of country-level contextual factors. Pain. 2022. 10.1097/j.pain.0000000000002557. 35027516 10.1097/j.pain.0000000000002557 PMC9198107

[6] Schofield P, Ruskin A, Gibson S. Pain in Older Adults. International Association for the Study of Pain. 2021. Availale from: https://www.iasp-pain.org/resources/fact-sheets/pain-in-older-adults/. Accessed 1 Nov 2025.

[7] Andrews P, Steultjens M, Riskowski J. Chronic widespread pain prevalence in the general population: a systematic review. Eur J Pain. 2018;22:5–18. 28815801 10.1002/ejp.1090

[8] Larsson B, Björk J, Börsbo B, Gerdle B. A systematic review of risk factors associated with transitioning from regional musculoskeletal pain to chronic widespread pain. Eur J Pain. 2012;16:1084–93. 22362638 10.1002/j.1532-2149.2012.00117.x

[9] Mansfield KE, Sim J, Jordan JL, Jordan KP. A systematic review and meta-analysis of the prevalence of chronic widespread pain in the general population. Pain. 2016. 10.1097/j.pain.0000000000000314. 26270591 10.1097/j.pain.0000000000000314 PMC4711387

[10] Khalatbari-Soltani S, Blyth FM. Socioeconomic position and pain: a topical review. Pain. 2022;163:1855–61. 35297800 10.1097/j.pain.0000000000002634

[11] Keralis JM. Pain and poverty: disparities by poverty level in the experience of pain-related interference. Pain Med. 2021;22:1532–8. 33527133 10.1093/pm/pnab030

[12] Zajacova A, Grol-Prokopczyk H, Fillingim R. Beyond Black vs White: racial/ethnic disparities in chronic pain including Hispanic, Asian, Native American, and multiracial US adults. Pain. 2022;163:1688–99. 35250011 10.1097/j.pain.0000000000002574 PMC9294074

[13] Hassan S, Muere A, Einstein G. Ovarian hormones and chronic pain: a comprehensive review. Pain. 2014. 10.1016/j.pain.2014.08.027. 25172822 10.1016/j.pain.2014.08.027

[14] Chand RR, Blyth FM, Khalatbari-Soltani S. Healthy dietary indices and noncancer pain: a systematic review of cross-sectional and longitudinal studies. Pain. 2023;164:e177–89. 36083185 10.1097/j.pain.0000000000002777

[15] Ferguson M, Svendrovski A, Katz J. Association between multimorbid disease patterns and pain outcomes among a complex chronic care population in Canada. J Pain Res. 2020;13:3045–57. 33244262 10.2147/JPR.S269648 PMC7685348

[16] Braden JB, Young A, Sullivan MD, Walitt B, LaCroix AZ, Martin L. Predictors of change in pain and physical functioning among post-menopausal women with recurrent pain conditions in the Women’s Health Initiative Observational Cohort. J Pain. 2012;13:64–72. 22208802 10.1016/j.jpain.2011.10.007 PMC3249604

[17] Jackson T, Thomas S, Stabile V, Han X, Shotwell M, McQueen K. Prevalence of chronic pain in low-income and middle-income countries: a systematic review and meta-analysis. Lancet. 2015;385:S10. 26313056 10.1016/S0140-6736(15)60805-4

[18] Inoue S, Kobayashi F, Nishihara M, Arai YC, P, Ikemoto T, et al. Chronic pain in the japanese community—prevalence, characteristics and impact on quality of life. PLoS ONE. 2015;10:e0129262. 26076135 10.1371/journal.pone.0129262 PMC4467865

[19] Raftery MN, Sarma K, Murphy AW, la De Harpe D, Normand C, McGuire BE. Chronic pain in the Republic of Ireland - community prevalence, psychosocial profile and predictors of pain-related disability: results from the Prevalence, Impact and Cost of Chronic Pain (PRIME) study, part 1. Pain. 2011;152:1096–103. 21450402 10.1016/j.pain.2011.01.019

[20] Dueñas M, Ojeda B, Salazar A, Mico JA, Failde I. A review of chronic pain impact on patients, their social environment and the health care system. J Pain Res. 2016;9:457–67. 27418853 10.2147/JPR.S105892 PMC4935027

[21] Bleicher K, Summerhayes R, Baynes S, Swarbrick M, Navin Cristina T, Luc H, et al. Cohort profile update: the 45 and Up Study. Int J Epidemiol. 2023;52:e92–101. 35604374 10.1093/ije/dyac104 PMC9908035

[22] Sax Institute. The 45 and Up Study. Wave 2 Data Book: First Follow-Up, 2012–2015. Sydney, Australia: Sax Institute; 2021. Available from: https://www.saxinstitute.org.au/wp-content/uploads/W2-databook-May2021.pdf.

[23] Sax Institute. Data and technical information. Sydney, Australia: Sax Institute. Available from https://www.saxinstitute.org.au/solutions/45-and-up-study/use-the-45-and-up-study/data-and-technical-information/.

[24] Stewart AL, Kamberg CJ. Physical Functioning Measures. In: Steward AL, Ware JE, editors. Measuring functioning and well-being: the medical outcomes study approach. Durham: Duke University Press; 1992. p. 86–101.

[25] Haley SM, McHorney CA, Ware JE Jr. Evaluation of the MOS SF-36 physical functioning scale (PF-10): I. unidimensionality and reproducibility of the Rasch item scale. J Clin Epidemiol. 1994;47:671–84. 7722580 10.1016/0895-4356(94)90215-1

[26] Kessler RC, Andrews G, Colpe LJ, Hiripi E, Mroczek DK, Normand SL, et al. Short screening scales to monitor population prevalences and trends in non-specific psychological distress. Psychol Med. 2002;32:959–76. 12214795 10.1017/s0033291702006074

[27] Andrews G, Slade T. Interpreting scores on the Kessler Psychological Distress Scale (K10). Aust N Z J Public Health. 2001;25:494–7. 11824981 10.1111/j.1467-842x.2001.tb00310.x

[28] Dominick CH, Blyth FM, Nicholas MK. Unpacking the burden: understanding the relationships between chronic pain and comorbidity in the general population. Pain. 2012;153:293–304. 22071318 10.1016/j.pain.2011.09.018

[29] Howe LD, Tilling K, Galobardes B, Lawlor DA. Loss to follow-up in cohort studies: bias in estimates of socioeconomic inequalities. Epidemiology. 2013;24:1–9. 23211345 10.1097/EDE.0b013e31827623b1 PMC5102324

[30] Powers J, Tavener M, Graves A, Loxton D. Loss to follow-up was used to estimate bias in a longitudinal study: a new approach. J Clin Epidemiol. 2015;68:870–6. 25700941 10.1016/j.jclinepi.2015.01.010

[31] Mealing NM, Banks E, Jorm LR, Steel DG, Clements MS, Rogers KD. Investigation of relative risk estimates from studies of the same population with contrasting response rates and designs. BMC Med Res Methodol. 2010. 10.1186/1471-2288-10-26. 20356408 10.1186/1471-2288-10-26 PMC2868856

[32] Eccleston C, Begley E, Birkinshaw H, Choy E, Crombez G, Fisher E, et al. The establishment, maintenance, and adaptation of high- and low-impact chronic pain: a framework for biopsychosocial pain research. Pain. 2023;164:2143–7. 37310436 10.1097/j.pain.0000000000002951 PMC10502876

[33] Von Korff M, DeBar LL, Krebs EE, Kerns RD, Deyo RA, Keefe FJ. Graded chronic pain scale revised: mild, bothersome, and high-impact chronic pain. Pain. 2020;161:651–61. 31764390 10.1097/j.pain.0000000000001758 PMC7097879

[34] Vitali D, Woolley CSC, Ly A, Nunes M, Lisboa LO, Keogh E, et al. How well can we measure chronic pain impact in existing longitudinal cohort studies? Lessons learned J Pain. 2025;26:104679. 39299445 10.1016/j.jpain.2024.104679

[35] Creed F. A review of the incidence and risk factors for fibromyalgia and chronic widespread pain in population-based studies. Pain. 2020;161:1169–76. 32040078 10.1097/j.pain.0000000000001819

[36] Costantini M, Viterbori P, Flego G. Prevalence of pain in italian hospitals: results of a regional cross-sectional survey. J Pain Symptom Manage. 2002;23:221–30. 11888720 10.1016/s0885-3924(01)00405-5

[37] Guo J, Fu M, Qu Z, Wang X, Zhang X. Risk factors associated with pain among community adults in Northwest China. J Pain Res. 2019;12:1957–69. 31308728 10.2147/JPR.S193773 PMC6615462

[38] Rustøen T, Wahl AK, Hanestad BR, Lerdal A, Paul S, Miaskowski C. Prevalence and characteristics of chronic pain in the general Norwegian population. Eur J Pain. 2004;8:555–65. 15531224 10.1016/j.ejpain.2004.02.002

[39] Thomas E, Peat G, Harris L, Wilkie R, Croft PR. The prevalence of pain and pain interference in a general population of older adults: cross-sectional findings from the North Staffordshire Osteoarthritis Project (NorStOP). Pain. 2004;110:361–8. 15275787 10.1016/j.pain.2004.04.017

[40] Blyth FM, March LM, Brnabic AJM, Jorm LR, Williamson M, Cousins MJ. Chronic pain in Australia: a prevalence study. Pain. 2001;89:127–34. 11166468 10.1016/s0304-3959(00)00355-9

[41] Sjøgren P, Ekholm O, Peuckmann V, Grønbæk M. Epidemiology of chronic pain in Denmark: an update. Eur J Pain. 2009;13:287–92. 18547844 10.1016/j.ejpain.2008.04.007

[42] Azevedo LF, Costa-Pereira A, Mendonça L, Dias CC, Castro-Lopes JM. Epidemiology of chronic pain: a population-based nationwide study on its prevalence, characteristics and associated disability in Portugal. J Pain. 2012;13:773–83. 22858343 10.1016/j.jpain.2012.05.012

[43] Cabral DMC, Bracher ESB, Depintor JDP, Eluf-Neto J. Chronic pain prevalence and associated factors in a segment of the population of Sao Paulo City. J Pain. 2014;15:1081–91. 25038400 10.1016/j.jpain.2014.07.001

[44] Mohanty SK, Ambade M, Upadhyay AK, Mishra RS, Pedgaonkar SP, Kampfen F, et al. Prevalence of pain and its treatment among older adults in India: a nationally representative population-based study. Pain. 2023;164:336–48. 36638306 10.1097/j.pain.0000000000002705

[45] Patel KV, Guralnik JM, Dansie EJ, Turk DC. Prevalence and impact of pain among older adults in the United States: findings from the 2011 National Health and Aging Trends Study. Pain. 2013. 10.1016/j.pain.2013.07.029. 24287107 10.1016/j.pain.2013.07.029 PMC3843850

[46] Portenoy RK, Ugarte C, Fuller I, Haas G. Population-based survey of pain in the United States: differences among white, African American, and Hispanic subjects. J Pain. 2004;5:317–28. 15336636 10.1016/j.jpain.2004.05.005

[47] Saastamoinen P, Leino-Arjas P, Laaksonen M, Lahelma E. Socio-economic differences in the prevalence of acute, chronic and disabling chronic pain among ageing employees. Pain. 2005. 10.1016/j.pain.2004.12.033. 15777862 10.1016/j.pain.2004.12.033

[48] Jimenez N, Garroutte E, Kundu A, Morales L, Buchwald D. A review of the experience, epidemiology, and management of pain among American Indian, Alaska Native, and Aboriginal Canadian Peoples. J Pain. 2011;12:511–22. 21330217 10.1016/j.jpain.2010.12.002 PMC3090505

[49] Fjeld MK, Årnes AP, Engdahl B, Morseth B, Hopstock LA, Horsch A, et al. Consistent pattern between physical activity measures and chronic pain levels: the Tromsø Study 2015 to 2016. Pain. 2022;164:838–47. 36083173 10.1097/j.pain.0000000000002773 PMC10026831

[50] Santos MC da Silva, Gabani FL, Dias DF, de Andrade SM, González AD, Loch MR, et al. Longitudinal associations of changes in physical activity and TV viewing with chronic musculoskeletal pain in Brazilian schoolteachers. PLoS One. 2020;15:e0234609. 10.1371/journal.pone.0234609 PMC7299367 32555745

[51] Khan JS, Hah JM, Mackey SC. Effects of smoking on patients with chronic pain: a propensity-weighted analysis on the Collaborative Health Outcomes Information Registry. Pain. 2019;160:2374–9. 31149975 10.1097/j.pain.0000000000001631 PMC6768701

[52] Picavet HSJ, Monique Verschuren WM, Groot L, Schaap L, van Oostrom SH. Pain over the adult life course: 15-year pain trajectories—The Doetinchem Cohort Study. Eur J Pain. 2019;23:1723–32. 31257661 10.1002/ejp.1450 PMC6790708

[53] Park JE, Ryu Y, Cho SI. The association between health changes and cessation of alcohol consumption. Alcohol Alcohol. 2017;52:344–50. 28430927 10.1093/alcalc/agw089 PMC5397877

[54] Lien W-H, Lien W-C, Kuan T-S, Wu S-T, Chen Y-T, Chiu C-J. Parkinson disease and musculoskeletal pain: an 8-year population-based cohort study. Pain. 2017;158:1234–40. 28328577 10.1097/j.pain.0000000000000904

[55] Giummarra MJ, Tardif H, Blanchard M, Tonkin A, Arnold CA. Hypertension prevalence in patients attending tertiary pain management services, a registry-based Australian cohort study. PLoS One. 2020;15:e0228173. 31978196 10.1371/journal.pone.0228173 PMC6980551

[56] van Hecke O, Hocking LJ, Torrance N, Campbell A, Padmanabhan S, Porteous DJ, et al. Chronic pain, depression and cardiovascular disease linked through a shared genetic predisposition: analysis of a family-based cohort and twin study. PLoS One. 2017;12:e0170653. 28225781 10.1371/journal.pone.0170653 PMC5321424

[57] Ryan CG, McDonough S, Kirwan JP, Leveille S, Martin DJ. An investigation of association between chronic musculoskeletal pain and cardiovascular disease in the Health Survey for England (2008). Eur J Pain. 2014;18:740–50. 24167109 10.1002/j.1532-2149.2013.00405.x

[58] Bruehl S, Olsen RB, Tronstad C, Sevre K, Burns JW, Schirmer H, et al. Chronic pain-related changes in cardiovascular regulation and impact on comorbid hypertension in a general population: the Tromsø study. PAIN. 2018;159(1):119–27. 10.1097/j.pain.0000000000001070. 10.1097/j.pain.0000000000001070 28953193

[59] Landefeld JC, Miaskowski C, Tieu L, Ponath C, Lee CT, Guzman D, et al. Characteristics and factors associated with pain in older homeless individuals: results from the health outcomes in people experiencing homelessness in older middle age (HOPE HOME) study. J Pain. 2017;18:1036–45. 28412229 10.1016/j.jpain.2017.03.011 PMC5581208

[60] Milani SA, Howrey B, Rodriguez MA, Samper-Ternent R, Wong R. Gender differences in activity-limiting pain trajectories over a 17-year period in the Mexican Health and Aging Study. Pain. 2022;163:e285–92. 33863866 10.1097/j.pain.0000000000002292 PMC8494819

[61] Reyes-Gibby CC, Aday LA, Todd KH, Cleeland CS, Anderson KO. Pain in aging community-dwelling adults in the United States: non-Hispanic Whites, non-Hispanic Blacks, and Hispanics. J Pain. 2007;8:75–84. 16949874 10.1016/j.jpain.2006.06.002 PMC1974880

[62] Arola HM, Nicholls E, Mallen C, Thomas E. Self-reported pain interference and symptoms of anxiety and depression in community-dwelling older adults: can a temporal relationship be determined? Eur J Pain. 2010;14:966–71. 20381389 10.1016/j.ejpain.2010.02.012

[63] de Heer EW, Gerrits MMJG, Beekman ATF, Dekker J, van Marwijk HWJ, de Waal MWM, et al. The association of depression and anxiety with pain: a study from NESDA. PLoS One. 2014;9:e106907. 25330004 10.1371/journal.pone.0106907 PMC4198088

[64] Søndergård S, Vaegter HB, Erlangsen A, Stenager E. Ten-year prevalence of mental disorders in patients presenting with chronic pain in secondary care: a register linkage cohort study. Eur J Pain. 2018;22:346–54. 28971547 10.1002/ejp.1124

[65] Ntani G, Coggon D, Felli VE, Harari F, Barrero LH, Felknor SA, et al. Patterns of change of multisite pain over 1 year of follow-up and related risk factors. Eur J Pain. 2022;26:1499–509. 35598315 10.1002/ejp.1978

[66] Aili K, Campbell P, Michaleff ZA, Strauss VY, Jordan KP, Bremander A, et al. Long-term trajectories of chronic musculoskeletal pain: a 21-year prospective cohort latent class analysis. Pain. 2021;162:1511–20. 33230006 10.1097/j.pain.0000000000002137 PMC8054552

[67] Dragioti E, Larsson B, Bernfort L, Levin LÅ, Gerdle B. A cross-sectional study of factors associated with the number of anatomical pain sites in an actual elderly general population: results from the PainS65+ cohort. J Pain Res. 2017;10:2009–19. 28883740 10.2147/JPR.S143060 PMC5574683

[68] Gureje O, Von Korff M, Simon GE, Gater R. Persistent pain and well-being. A World Health Organization study in primary care. JAMA. 1998;280:147–51. 9669787 10.1001/jama.280.2.147

[69] Jakobsson U, Klevsgård R, Westergren A, Hallberg IR. Old people in pain: a comparative study. J Pain Symptom Manage. 2003;26:625–36. 12850645 10.1016/s0885-3924(03)00145-3

[70] Gatchel RJ, Peng YB, Peters ML, Fuchs PN, Turk DC. The biopsychosocial approach to chronic pain: scientific advances and future directions. Psychol Bull. 2007;133:581–624. 17592957 10.1037/0033-2909.133.4.581

