# Supplementary material for: Pain and high-impact pain in community-dwelling older adults in Australia and relation to sociodemographic and health-related factors, including physical disability, psychological distress, and quality of life
Source: BMC Med. 2026 Jan 22;24:70. doi: 10.1186/s12916-026-04642-0 (PMC12870111; doi:10.1186/s12916-026-04642-0)
Supplement: Supplementary file 1 — Additional file 1. [file 12916_2026_4642_MOESM1_ESM.pdf]

Additional File 1: Supplementary Tables and Figures

Table of Contents

Table S1. Assessment method for bodily pain and high-impact pain in the 45 & Up Study 2

Table S2. Assessment method for sociodemographic, behavioural, health-related factors, and person-centred outcomes in the 45 & Up Study....3

Table S3. Definition of impact of pain....9

Figure S1. Levels of bodily pain and impact of pain reported by lower back pain....10

Figure S2. Levels of bodily pain and impact of pain by age and sex....11

Table S4. Prevalence of bodily pain and high-impact pain by age and sex....12

Table S5. Prevalence of chronic conditions, considered together with lower back pain....13

Table S6. Prevalence of bodily pain and high-impact pain by various combinations of chronic conditions....14

Table S7. Prevalence of bodily pain and high-impact pain by number of chronic conditions....15

Supplementary Tables/Figures

Table S1. Assessment method for bodily pain and high-impact pain in the 45 & Up Study

Outcome	***Study wave***	***Question***	***Response options***	***Categorisation***
Bodily pain	Follow up questionnaire	How much bodily pain have you had during the past 4 weeks?	• None<br>• Very mild<br>• Mild<br>• Moderate<br>• Severe<br>• Very severe	• Bodily pain -Yes: 'moderate', 'severe' or 'very severe'.<br>• Bodily pain - No: "none", "very mild" or "mild".
High-impact pain	Follow up questionnaire	1. How much bodily pain have you had during the past 4 weeks?<br>○ None<br>○ Very mild<br>○ Mild<br>○ Moderate<br>○ Severe<br>○ Very severe<br>2. During the past 4 weeks, how much did pain interfere with your normal work (including both work outside the home and housework)?<br>○ Not at all<br>○ A little bit<br>○ Quite a bit<br>○ Moderately<br>○ Extremely	Response options for Question 1<br>• None<br>• Very mild<br>• Mild<br>• Moderate<br>• Severe<br>• Very severe<br><br>Response options for Question 2<br>• Not at all<br>• A little bit<br>• Quite a bit<br>• Moderately<br>• Extremely	• High-impact pain: any pain interfering 'moderately' or 'extremely' with normal work<br>• Bothersome pain: at least 'moderate' pain interfering 'a little' or 'quite a bit' with normal work)<br>• Low-impact pain: (any pain 'not at all' interfering with normal work) OR ('very mild' or 'mild' pain interfering 'a little' or 'quite a bit' with normal work)<br>• No impact of pain (no pain or pain interference)<br><br>Further details are provided in Table S3.

Baseline and follow-up questionnaires can be viewed at https://www.saxinstitute.org.au/solutions/45-and-up-study/use-the-45-and-up-study/data-and-technical-information/

Table S2. Assessment method for sociodemographic, behavioural, health-related factors, and person-centred outcomes in the 45 & Up Study

Variable	Study wave	Question	Response options	Categorisation
Age	Baseline questionnaire	• What is your date of birth?<br>• What is today's date?	Continuous	Calculated based on self-reported date of birth and today's date<br>• 45–64 years<br>• 65–79 years<br>• ≥80 years
Sex	N/A	N/A (sex at the time of original recruitment as recorded by Medicare Australia)	N/A	• Male<br>• Female
Education	Baseline questionnaire	What is the highest qualification you have completed?	• No school certificate or other qualifications<br>• School or intermediate certificate (or equivalent)<br>• Higher school or leaving certificate (or equivalent)<br>• Trade/apprenticeship (e.g., hairdresser, chef)<br>• Certificate/diploma (e.g., childcare, technician)<br>• University degree or higher	• No school certificate<br>• Certificate/diploma/trade<br>• University degree
Region of residence	N/A	N/A (derived for each participant's postcode of residence at the time of original recruitment as recorded by Medicare Australia)	N/A (Remoteness areas, based on enhanced measures of remoteness (ARIA+) developed by the National Key Centre for Social	• Major city<br>• Inner regional<br>• Outer regional<br>• Remote/very remote

			Applications of Geographic Information Systems)	
Country of birth	Baseline questionnaire	In which country were you born?	A list of countries, including Australia, UK, Ireland, Italy, etc., and other (please specify)	• Australia<br>• Not Australia
Body mass index (BMI)	Follow up questionnaire	• How tall are you without shoes?<br>• About how much do you weigh?	Continuous	Calculated based on self-reported height and weight<br>• Underweight (15–<18.5)<br>• Normal weight (18.5–<25)<br>• Overweight (25–<30)<br>• Obese (≥30–50)
Physical activity	Follow up questionnaire	How many TIMES did you do each of these activities LAST WEEK?<br>How many hours and minutes did you do each of these activities LAST WEEK?<br>-Times and duration of physical activity weighted by intensity.	• Walking continuously, for at least 10 minutes<br>• Vigorous physical activity<br>• Moderate physical activity	• First tertile<br>• Second tertile<br>• Third tertile
Smoking status	Follow up questionnaire	-Have you been a regular smoker?<br>-If no, how old were you when you stopped smoking regularly?	-Yes/No<br><br>-continuous	• Current smoker<br>• Past smoker<br>• Never smoker
Alcohol consumption	Follow up questionnaire	About how many alcoholic drinks do you have each week?	Continuous	• 0<br>• 1–14<br>• ≥15
Health conditions	Follow up questionnaire	Has a doctor ever told you that you have…	Tick boxes for:<br>• Skin cancer (not melanoma)	• Yes<br>• No

			• Melanoma<br>• Breast cancer<br>• Other cancer (please specify)<br>• Heart disease<br>• Stroke<br>• Blood clotting problems<br>• High blood-pressure (when not pregnant)<br>• Stroke<br>• Diabetes<br>• Blood clot (thrombosis)<br>• Asthma<br>• Hay fever<br>• Osteoarthritis<br>• Depression<br>• Anxiety<br>• Parkinson's disease<br>• None of these	Cardiovascular disease was based on any of heart disease, stroke or blood clot.<br><br>For asthma, there were two versions of the question. The first version is "has a doctor ever told you that you have: asthma or hay fever?". The second version is asthma only. We used version 2 here, while the answer options are yes, no, and 999. Participants who only answered version 1 of the question were coded as 999. We regard these participants as those with missing values for asthma.
Self-rated health	Follow up questionnaire	In general, how would you rate your<br>-overall health?	• Excellent<br>• Very good<br>• Good<br>• Fair<br>• Poor	Same as response options
Self-rated quality of life	Follow up questionnaire	In general, how would you rate your<br>-quality of life?	• Excellent<br>• Very good<br>• Good<br>• Fair<br>• Poor	Same as response options
Physical functioning limitations	Follow up questionnaire	Does your health now LIMIT YOU in any of the following activities?	• YES limited a lot (score=1)	Medical Outcomes Study Physical Functioning (MOS-PF) score is the sum of 10 individual

		1. Vigorous activities<br>2. Moderate activities<br>3. Lifting or carrying shopping<br>4. Climbing several flights of stairs<br>5. Climbing one flight of stairs<br>6. Walking one kilometre<br>7. Walking half a kilometre<br>8. Walking 100 metres<br>9. Bending, kneeling or stooping<br>10. Bathing or dressing yourself	• YES limited a little (score=2)<br>• NO not limited at all (score=3)	item scores, rescaled to range from 0 to 100. MOS-PF scores are categorised as:<br>• No limitation MOS-PF=100<br>• Minor limitations 90≤MOS-PF<100<br>• Moderate limitations 60≤MOS-PF<90<br>• Severe limitations 0≤MOS-PF<60
Psychological distress	Follow up questionnaire	During the past four weeks, about how often did you feel:<br>1. Tired for no good reason?<br>2. Nervous?<br>3. So nervous that nothing could calm you down?<br>4. Hopeless?<br>5. Restless or fidgety?<br>6. So restless that you could not sit still?<br>7. Depressed?<br>8. That everything was an effort?<br>9. So sad that nothing could cheer you up?	• None of the time (score=1)<br>• A little of the time (score=2)<br>• Some of the time (score=3)<br>• Most of the time (score=4)<br>• All of the time (score=5)	Kessler-10 (K10) score for psychological distress is the sum of 10 individual item scores. K10 score range from 10 to 50, and are categorised as:<br>• Low distress 10≤K10<16<br>• Moderate distress 16≤K10<22<br>• High distress 22≤K10≤50

		10. Worthless?		
Annual household income	Follow up questionnaire	What is your usual yearly HOUSEHOLD income before<br>tax, from all sources? (include wages, benefits, pensions,<br>superannuation etc.)	• less than $5,000<br>• $5,000 - $9,999<br>• $10,000 - $19,999<br>• $20,000 - $29,999<br>• $30,000 - $39,999<br>• $40,000 - $49,999<br>• $50,000 - $59,999<br>• $60,000 - $69,999<br>• $70,000 - $79,999<br>• $80,000 - $89,999<br>• $90,000 - $119,999<br>• $120,000 - $149,999<br>• $150,000 or more<br>• I would rather not answer this question	Annual household income is categorised as:<br>• <20K<br>• 20K–40<br>• 40K–70<br>• ≥70K
Private health insurance	Follow up questionnaire	Which of the following do you have (excluding Medicare)? (shade all that apply)	• private health insurance – with extras<br>• private health insurance – without extras<br>• Department of Veterans' Affairs White or Gold Card<br>• health care concession card<br>• none of these	• No private health insurance<br>• Hospital/DVA
Regular medication	Follow up questionnaire	Have you taken any medications, vitamins or supplements for most of the last 4 weeks? (Yes/No)<br>If yes, did you take:	• Paracetamol<br>• Paracetamol with codeine<br>• Aspirin for the heart<br>• Aspirin for other reasons	• Paracetamol without codeine<br>• Paracetamol with codeine<br>• Aspirin for non-cardiac reasons<br>• None of the above
Received cancer	Follow up questionnaire	In the last month have you been treated for: (if YES, shade the box and give your	Tick box for:<br>• Cancer	• Yes<br>• No

treatment in last month		age when the treatment started)		
Lower back pain	Follow up questionnaire	In the past 4 weeks, have you had pain in your lower back?	• Yes<br>• No	• Yes<br>• No

Baseline and follow-up questionnaires can be viewed at https://www.saxinstitute.org.au/solutions/45-and-up-study/use-the-45-and-up-study/data-and-technical-information/

Table S3. Definition of impact of pain

BODILY PAIN[a]		PAIN INTERFERING WITH NORMAL WORK[b]		
Finer classification	Binary classification	Not at all	A little bit or quite a bit	Moderately or extremely
None	Bodily pain - No	No impact (n=32,309)	Not applicable	Not applicable
Very mild or mild	Bodily pain - No	Low-impact (n=36,518)	Low-impact (n=23,406)	High-impact (1,115)
Moderate, severe or very severe	Bodily pain - Yes	Low-impact (n=4,398)	Bothersome (n=22,246)	High-impact (17,107)

[a]Based on the question "How much bodily pain have you had during the past 4 weeks?"; response options: None, Very mild, Mild, Moderate, Severe, Very severe.

[b]Based on the question "During the past 4 weeks, how much did pain interfere with your normal work (including both work outside the home and housework)?"; response options: Not at all, A little bit, Quite a bit, Moderately, Extremely.

Impact of pain was categorised as no impact (n=32,309), low-impact (n=64,322), bothersome (n=22,246) or high-impact (18,222).

Figure S1. Levels of bodily pain and impact of pain reported by lower back pain

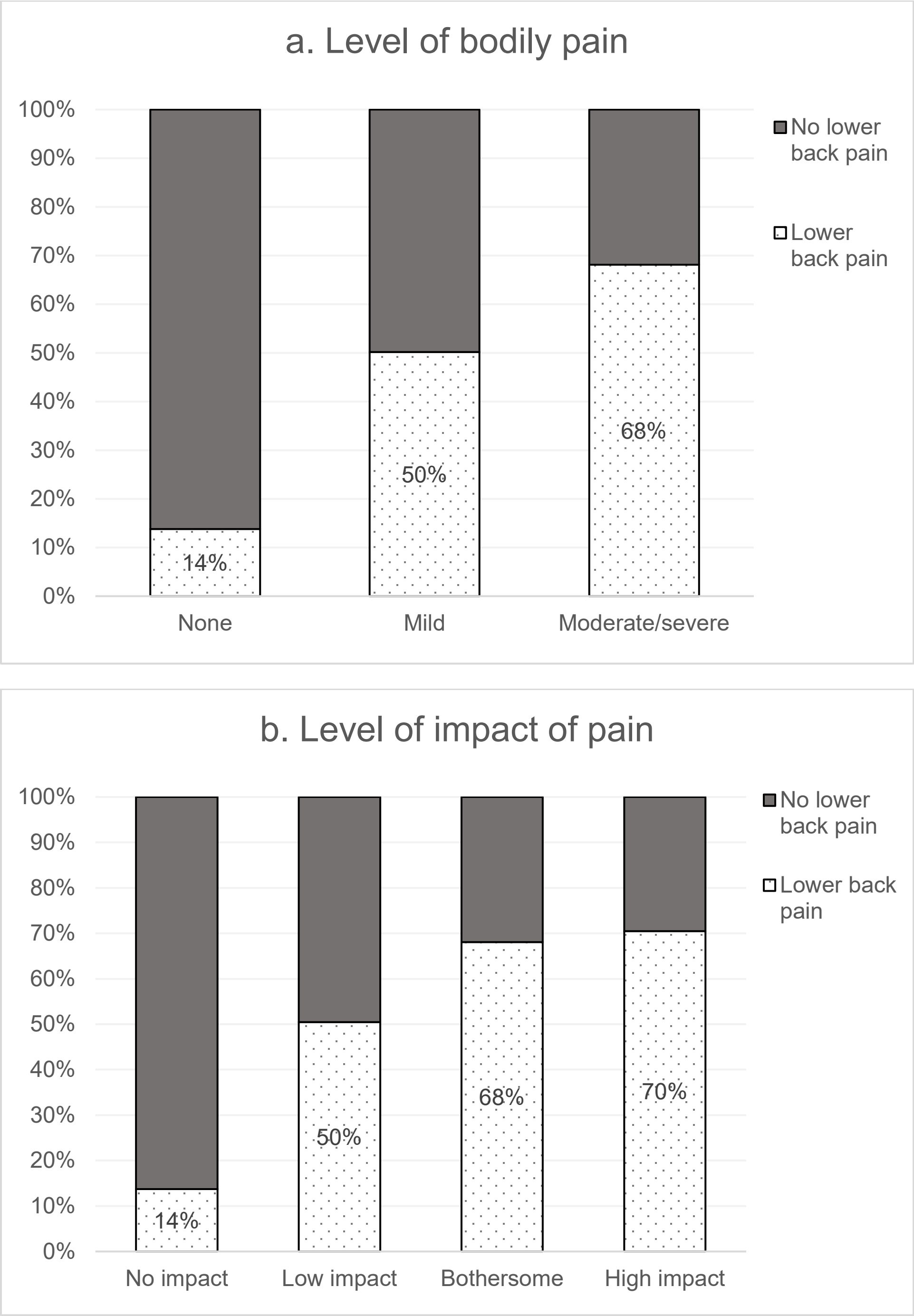

Figure S2. Levels of bodily pain and impact of pain by age and sex

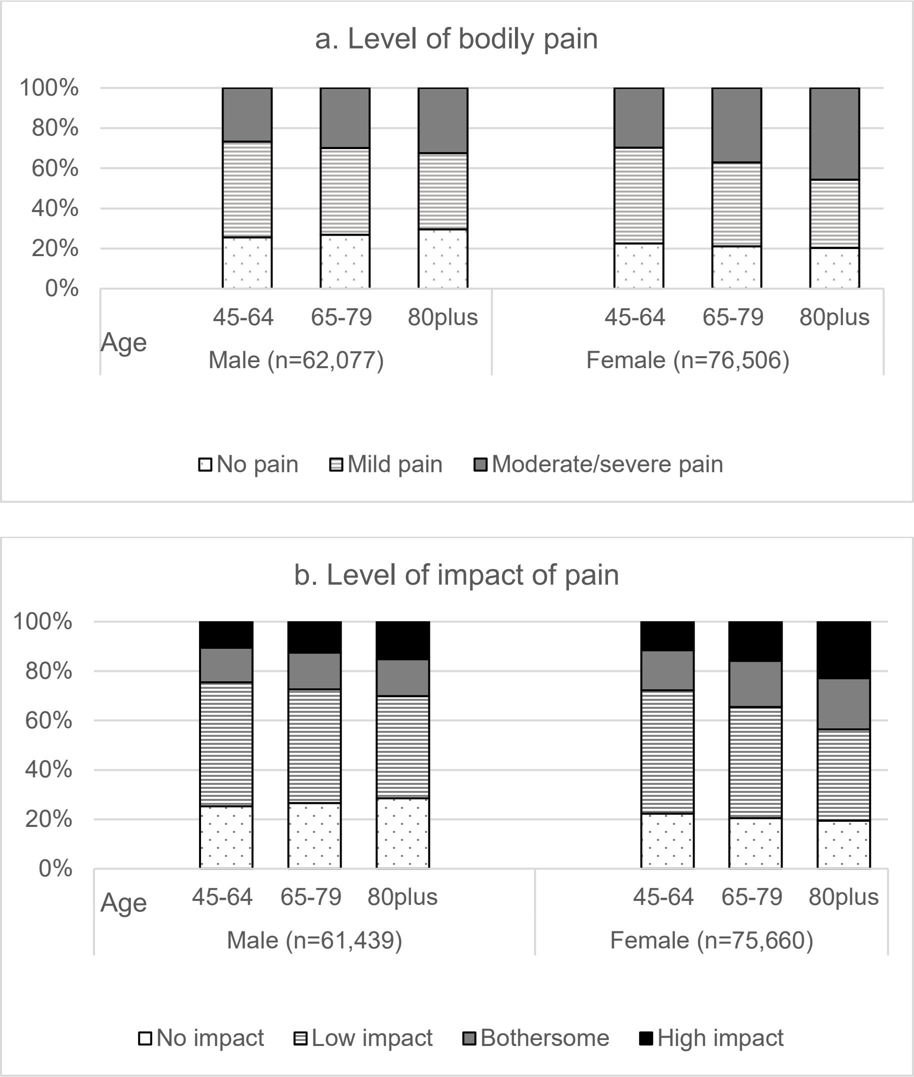

Table S4. Prevalence of bodily pain and high-impact pain by age and sex

Age (years)	Male		Female		Total	
	%	PR (95% CI)	%	PR (95% CI)	%	PR (95% CI)
**Prevalence of bodily pain**						
45–64	26.7	1.00	29.8	1.00	28.6	1.00
65–79	30.0	1.13 (1.1–1.16)	37.1	1.25 (1.22–1.27)	33.7	1.20 (1.18–1.22)
80plus	32.4	1.22 (1.17–1.26)	45.7	1.53 (1.49–1.58)	38.7	1.39 (1.36–1.42)
**Prevalence of high-impact pain**						
45–64	10.6	1.00	11.6	1.00	11.2	1.00
65–79	12.5	1.18 (1.13–1.24)	15.8	1.37 (1.31–1.42)	14.2	1.29 (1.26–1.33)
80plus	15.2	1.44 (1.35–1.53)		1.97 (1.87–2.07)	18.8	1.73 (1.66–1.79)

PR estimates are crude estimates for males and females, and sex-adjusted estimates for the total study population. CI: confidence interval; PR: prevalence ratio adjusted for age and sex.

Table S5. Prevalence of chronic conditions, considered together with lower back pain

	n	%
Ever diagnosed with cardiovascular disease, cancer, diabetes, Parkinson's disease, asthma, osteoarthritis, depression or anxiety	87,756	62%
Lower back pain in the past four weeks	67,078	47%
No chronic conditions listed above or lower back pain at baseline	32,891	23%

Table S6. Prevalence of bodily pain and high-impact pain by various combinations of chronic conditions

Chronic condition		n	% with bodily pain	% with high-impact pain
Osteoarthritis				
	Osteoarthritis alone	9094	49	20
	Osteoarthritis and cancer	4963	61	31
	Osteoarthritis and depression	5510	71	38
	Osteoarthritis and anxiety	4116	70	37
	Osteoarthritis and diabetes	3283	69	38
	Osteoarthritis and any of cancer, depression, anxiety, diabetes	12301	65	34
	Osteoarthritis and any of cancer, depression, anxiety, diabetes, CVD, Parkinson's disease, asthma	17685	63	32
	No osteoarthritis	115534	26	10
Cancer				
	Cancer alone	8346	24	9
	Cancer and depression	3446	54	28
	Cancer and anxiety	2481	53	27
	Cancer and diabetes	2821	48	24
	Cancer and any of depression, anxiety, diabetes	6580	49	24
	Cancer and any of depression, anxiety, diabetes, CVD, Parkinson's disease, asthma	12141	44	21
	No cancer	120049	31	13
Depression				
	Depression alone	4671	32	13
	Depression and anxiety	9021	53	27
	Depression and diabetes	2704	63	35
	Depression and any of anxiety, diabetes	10533	54	28
	Depression and any of anxiety, diabetes, CVD, Parkinson's disease, asthma	14328	53	27
	No depression	121190	29	12
Anxiety				
	Anxiety alone	2676	26	10
	Anxiety and diabetes	1743	62	34
	Anxiety and any of diabetes, CVD, Parkinson's disease, asthma	6716	56	28
	No anxiety	126933	30	12
Diabetes				
	Diabetes alone	4474	28	11
	Diabetes and any of CVD, Parkinson's disease, asthma	6251	51	27
	No diabetes	128110	31	12

Table S7. Prevalence of bodily pain and high-impact pain by number of chronic conditions

	a. Bodily pain		b. High-impact pain	
	% (n/N)	PR (95% CI)	% (n/N)	PR (95% CI)
**Considering five chronic conditions with known relationship to pain**[1]				
No chronic condition	22.6 (17780/78688)	1	8 (6261/77855)	1
One chronic condition	39.4 (18044/45757)	1.7 (1.67–1.73)	17.1 (7754/45257)	2.04 (1.98–2.11)
Two or more chronic conditions	58.8 (8314/14138)	2.49 (2.45–2.54)	30.1 (4207/13987)	3.48 (3.36–3.61)
**Considering three chronic conditions with no specific relationship to pain**[2]				
No chronic condition	27.9 (26468/94828)	1	10.9 (10223/93898)	1
One chronic condition	38.9 (15210/39051)	1.36 (1.34–1.38)	17.5 (6761/38566)	1.53 (1.49–1.57)
Two or more chronic conditions	52.3 (2460/4704)	1.78 (1.73–1.83)	26.7 (1238/4635)	2.24 (2.13–2.36)
**Considering all eight chronic conditions**[3]				
No chronic condition	19.5 (10390/53258)	1	6.5 (3407/52726)	1
One chronic condition	31 (14201/45784)	1.57 (1.53–1.6)	12.1 (5491/45269)	1.82 (1.74–1.89)
Two or more chronic conditions	49.4 (19547/39541)	2.45 (2.4–2.5)	23.8 (9324/39104)	3.47 (3.34–3.61)

[1]Chronic conditions with known relationship to pain – cancer, diabetes, osteoarthritis, anxiety, and depression.
[2]Chronic conditions with no specific relationship to pain - CVD, Parkinson's and asthma.
[3]All eight chronic conditions - cancer, diabetes, osteoarthritis, anxiety, depression, cardiovascular disease, Parkinson's disease and asthma.
CI: confidence interval; PR: prevalence ratio adjusted for age and sex.
